# Adhesion of 3D-Printed Versus Milled Resin Posts to Composite Resin Core Build-Up Material: Influence of Surface Treatments

**DOI:** 10.3390/polym17121711

**Published:** 2025-06-19

**Authors:** Khalid K. Alanazi, Ali Robaian Alqahtani, Abdullah Mohammed Alshehri, Abdullah Ali Alqahtani, Abdulellah Almudahi, Omar Abdulaziz Al-Mansour, Nawaf Abdullah Al-Harbi, Sultan Sahman Abdulrahman Alqahtani, Eman Mohamed Raffat Hussein, Tarek Ahmed Soliman

**Affiliations:** 1 Conservative Dental Sciences, College of Dentistry, Prince Sattam Bin Abdulaziz University, Al-Kharj 16278, Saudi Arabia; ali.alqahtani@psau.edu.sa (A.R.A.); am.alshehri@psau.edu.sa (A.M.A.); aa.alqahtany@psau.edu.sa (A.A.A.); a.almudahi@psau.edu.sa (A.A.); 2 Dental Hospital, College of Dentistry, Prince Sattam Bin Abdulaziz University, Al-Kharj 16278, Saudi Arabia; oalmansuor25@gmail.com (O.A.A.-M.); nawaf228011@gmail.com (N.A.A.-H.); 3 Clinical Endodontics, Ministry of Health, Aseer Health Sector, Specialized Dental Center, Abha P.O. Box 2653, Saudi Arabia; drsultan607@hotmail.com; 4 Prosthetic Dentistry Department, Faculty of Dentistry, Benha National University, Benha 13511, Egypt; emanraffat70@gmail.com; 5 Dental Biomaterials Department, Faculty of Dentistry, Mansoura University, Mansoura 35516, Egypt

**Keywords:** bond strength, core material, 3D printing, surface treatments

## Abstract

**Background:** There are very few studies in literature concerning the bonding between 3D-printed resin posts and the core build-up material. This study aimed to evaluate and compare the adhesion of 3D-printed and milled resin posts to composite resin core build-up material following different surface treatments. **Methods:** Three types of resin posts were utilized in this study: ready-made glass-reinforced fiber post (3M ESPE, Germany), milled PEEK POST (Bredent, Germany), and 3D-printed resin post (CROWNTEC, Saremco Dental AG, Switzerland). Each type of post was categorized into three groups based on surface treatments: C: untreated surfaces; SB: Air abrasion with 50 μm aluminum oxide particles was applied to the posts’ surfaces.; HO: the posts’ surfaces were immersed in 30% H_2_O_2_ for 5 min. A dual-cured composite resin (Grandio DC; VOCO) was utilized for core build-up in each group to evaluate adhesion through the push-out bond strength test. The modes of failure were analyzed, and the surface morphology of the post was characterized using SEM. Data were analyzed using a two-way analysis of variance (ANOVA) along with Tukey’s test. **Results:** The two-way ANOVA indicated a significant effect for surface treatment (F = 583.54, *p* < 001), post type (F = 79.96, *p* < 0.001), and their interactions (F = 265.74, *p* < 0.001). Regarding 3D-printed resin post, 30% H_2_O_2_ for 5 min recorded the highest statistically significant bond strength value (13.11 ± 1.61) compared to other groups. Regarding the milled PEEK post, the air particle abrasion recorded the highest statistically significant value (23.88 ± 1.66) compared to other groups. Adhesive failure was the predominant failure type, with an occurrence rate of 70.35%. Mixed failure was noted in 24.07% of the cases, with a significant prevalence in the PEEK post within the air particle abrasion group (58.3%). Cohesive failure was noted in 5.54% of cases, with a significant prevalence in the air particle abrasion group, occurring at rates of 16.6% in the resin fiber post group and 33.3% in PEEK posts. **Conclusions:** Air particle abrasion significantly improved the push-out bond strength of milled PEEK posts, but it did not have a similar effect on the 3D-printed resin posts. The application of 30% H_2_O_2_ for 5 min to 3D-printed resin post enhanced the adhesion to core build-up material. The manufacturing method of posts, the surface treatments utilized, and their interactions affect the interfacial bond strength between posts and the composite resin core build-up material.

## 1. Introduction

Teeth that have undergone endodontic treatment and have a coronal loss of over 50% are susceptible to shearing chewing forces and frequently necessitate a post-and-core. The effectiveness of post-retained restorations relies on the establishment of a strong bond at the post-core interface [1,2]. The bonding effectiveness between posts and core build-up material may be influenced by the surface treatments applied to the post, the type of post manufactured, and the composite resin core material [3,4].

In the context of post/core restorations, a number of different surface treatments have been suggested as potential ways to enhance the adherence of composite resin cores to posts [1,2]. Air particle abrasion using aluminum oxide is a method designed to improve the bond between dental posts and composite resin core materials. It involves roughening the post surface to increase surface area, which increases mechanical retention [5,6]. To mitigate the risk of post damage caused by air particle abrasion, alternative chemical treatments such as hydrogen peroxide have been suggested to enhance the adhesion of post-retained restorations. H_2_O_2_ is frequently employed in dental practice, effectively eliminating the surface layer of epoxy resin, and is easy and safe to use [7,8].

Glass-reinforced fiber posts are frequently utilized in dental procedures to enhance support and retention for the restoration. When compared to dentin (8.6 GPa), the elastic modulus is around three times higher, ranging from 45.7 to 53.8 GPa [9,10]. Interfacial detachment, fiber fracture, and resin matrix cracking represent mechanisms that contribute to the failure of adhesion between fiber posts and core build-up materials [3,4]. The advancement of digital dentistry in clinical practice has resulted in the extensive implementation of CAD/CAM techniques. Two primary approaches exist in CAD/CAM. The initial method of digital design and manufacturing mainly consisted of subtractive milling from a pre-polymerized block of the restorative material [11,12]. While the development holds significant importance, a primary drawback linked to subtractive CAD/CAM methods is the considerable material waste during the milling process [13,14]. Recent developments in CAD/CAM technologies for dental resins have facilitated the rise of additive manufacturing methods, known as 3D printing. Restorations are developed in incremental layers that align with the intended shape, thus reducing material waste. 3D printing presents several benefits over fabrication using milling techniques. The lack of cutting tools in 3D printing facilitates unrestricted movement, leading to enhanced marginal fit. This method is cost-effective and minimizes waste [11,12,13,14].

Numerous investigations in the literature have focused on the bond strength of CAD/CAM milled posts and glass fiber posts. There have been a number of previous studies that have investigated the effect of surface treatments on the adherence of milled PEEK posts to core-build-up materials. The primary factor contributing to the bond between PEEK and resin materials is attributed to micromechanical locking, which occurs due to the penetration of resin into fissures or pores on the PEEK surface [15,16,17]. Additionally, recent studies have focused on the mechanical performance of 3D-printed resins [12,18,19,20,21]. Nevertheless, there are limited studies in the literature regarding the bonding between 3D-printed resin posts and the core build-up material. This study aimed to evaluate and compare the adhesion of a 3D-printed and milled resin post to composite resin core build-up material following different surface treatments. The null hypothesis stated that (1) there is no significant difference in bond strength among the fiberglass, milled, and 3D-printed resin post systems, and (2) surface treatments had no significant effect on the bond strength of the different types of posts.

## 2. Materials and Methods

### 2.1. Materials and Study Design

**Figure 1:**
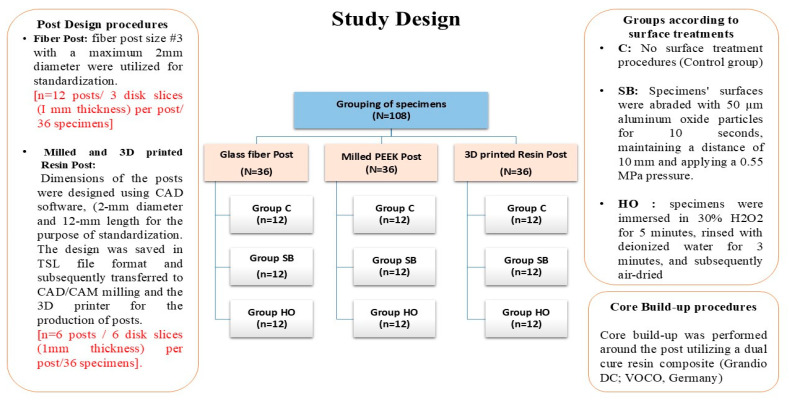
Study design and specimens’ grouping.

Three resin posts were utilized in this study: ready-made glass-reinforced fiber post (3M ESPE, Seefeld, Germany), Milled PEEK POST (Bredent, Senden, Germany), and 3D-printed resin post (CROWNTEC, Saremco Dental AG, Rebstein, Switzerland). Each type of post was classified into three groups according to surface treatments. The core build-up was performed around the post utilizing a dual-cure resin composite (Grandio DC; VOCO, Cuxhaven, Germany). A total of 12 specimens per group was required to attain a power of 0.95 at a 5% significance level. The materials used in this study and their manufacturers are presented in Table 1. The study design is presented in Figure 1.

### 2.2. Specimens’ Preparation

Ready-made fiberglass reinforced composite post (size #3) was sectioned to obtain a maximum 2 mm diameter for standardization [*n* = 12 posts/3 disk slices (1 mm thickness) per post/36 specimens]. Concerning the milled and 3D-printed resin post, the dimensions of the post were designed using CAD software (Meshmixer 3.5.0; Autodesk, San Francisco, CA, USA), establishing a diameter of 2 mm and a length of 12 mm for the purpose of standardization [*n* = 6 posts/6 disk slices (1 mm thickness) per post/36 specimens]. The design was saved in STL file format and then transferred to a milling machine (Ceramill Motion 2, Amann Girrbach AG, Austria) and a desktop 3D printer (Asiga Max UV printer, Australia) to produce six milled PEEK and six 3D printed posts. Six posts were milled from PEEK material according to the specified dimensions. Milling tools (Roto RFID, Amann Girrbach, Koblach, Austria) were utilized to achieve the desired shape. Tungsten carbide burs (1958-012 Jet Tungsten Carbide Bur, Kerr, Brea, CA, USA) were utilized to separate the specimens from the disc and to refine the attachment points. The 3D-printed specimens were produced in a single batch to ensure consistency in printing parameters and to eliminate variability. The 3D printer was pre-calibrated and tested to assure dimensional accuracy and process stability during printing. Following printing, the specimens were visually and dimensionally checked to ensure design compliance. No significant defects or inconsistencies were observed among the specimens. The 3D-printed post was laminated parallel to its long axis [11,18]. The printed specimens were carefully removed from the printing platform and ultrasonically cleaned with 90% isopropyl alcohol and then completely dried and cured for 7 min using a post-curing machine (Otoflash G171-N2, NK Optik GmbH, Baierbrunn, Germany). All specimens were finished using silicon carbide papers of varying grit sizes (600–2000 grits) while being cooled with water, followed by a 3-min ultrasonic washing in deionized water to achieve a uniform standard. The specimen dimensions were confirmed to be accurate with a digital caliper.

### 2.3. Grouping of Specimens

Posts were immersed in a 70% alcohol ultrasonic bath for 10 min to eliminate any superficial contaminants. Each type of post was categorized into three groups based on surface treatments: C: untreated surfaces; SB: Air abrasion with particles 50 µm was applied to the posts’ surfaces for 10 s at a distance of 10 mm and a pressure of 0.55 MPa. This was followed by 20 s of air drying [5,6,11]; HO: The posts’ surfaces were immersed in 30% H_2_O_2_ for 5 min, rinsed with deionized water for 3 min, and subsequently air-dried [7,8]. Following the manufacturer’s instructions, the surface of the post was treated with Single Bond Universal (3M ESPE, St. Paul, MN, USA) using a micro brush. After allowing the post to air dry gently for 60 s, it was then light polymerized for 20 s (Elipar Free light 2, 3M ESPE, 1226 mW/cm^2^).

### 2.4. Core Build-Up Procedure

A dual-cure composite material was utilized for the core build-up. Each post was placed at a right angle and held in place with a drop of adhesive wax [16,22]. A cylindrical plastic matrix with a diameter of 10 mm was then used to enclose the post. The core was created using the incremental technique and cured for 20 s using a LED light (Elipar Freeligh 2, 3M ESPE, 1226 mW/cm^2^), adhering to the manufacturer’s instructions, from the matrix’s opening and through the post. The curing tip position was as close as possible to the resin surface according to the recommended manufacturer instructions (1–2 mm) and perpendicular to the surface of the resin throughout the exposure cycle. The curing started after a 20–40 s delay to allows the chemical cure to initiate. An additional 20 s of curing from the bottom is recommended to guarantee optimum polymerization. The post-core assembly was sectioned using a low-speed diamond saw with water cooling, yielding six disk specimens, each with a thickness of 1 mm. All specimens were immersed in standardized conditions at 37 °C and 100% humidity for 24 h before bond strength testing.

### 2.5. Push-Out Bond Strength Test

**Figure 2:**
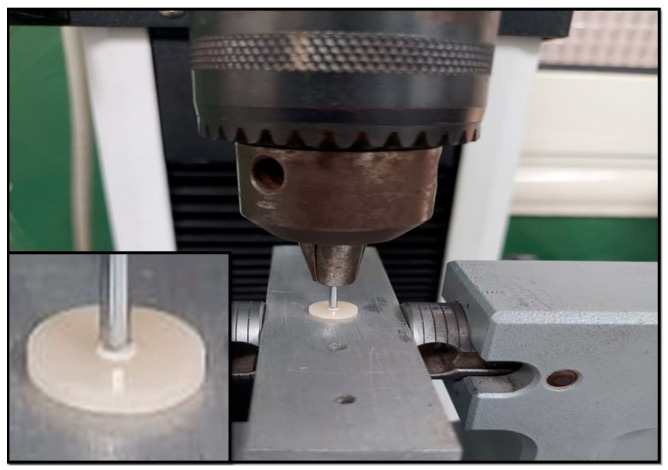
Push-out bond strength testing.

The push-out bond strength was assessed using a universal testing machine (Instron 5965, Canton, MA, USA). Specimens were secured in the lower jaw of the machine. A cylindrical plunger with a diameter of 1 mm was positioned centrally on the disk specimen. The specimen was subjected to compressive loading at a crosshead speed of 0.5 mm/min. (Figure 2). Bond strength is calculated in MPa by dividing the failure load by the bonding area (N/mm^2^). A = 2r × π × h provides the bonding area for each post, r is the post radius, π is approximately 3.14, and h is the thickness of the post slice [23]. Using a stereomicroscope at 40× magnification. Failure modes were classified as adhesive failure between the post and core materials, cohesive failure within the post or core material, and mixed failure.

### 2.6. Scanning Electron Microscopy

A scanning electron microscope was used to evaluate the morphological characteristics of posts after different surface treatments. The specimens underwent ultrasonic cleaning prior to a 2-min soak in 96% ethanol, followed by air drying. Every specimen received sputter-coating with gold and was subsequently examined under a microscope.

### 2.7. Statistical Analysis

Shapiro–Wilk and Levene’s tests confirmed that the assumptions of normality and homogeneity of variance were fulfilled. A two-way analysis of variance was utilized to assess the effects of post type manufacturer and surface treatments, as well as their interaction on push-out bond strength. To identify statistically significant differences between groups, post-hoc analyses were conducted using Tukey’s significant difference test. A significant level of 5% was set for all statistical tests.

## 3. Results

The two-way analysis of variance (ANOVA) table showed a significant impact of surface treatment (F = 583.54, *p* < 0.001), type of post (F = 79.96, *p* < 0.001), and their interaction (F = 265.74, *p* < 0.001) (Table 2). Table 3 displays the bond strength values for all groups. The SB group in the PEEK post demonstrated the highest bond strength (23.88 ± 1.51 MPa), whereas the lowest bond strength was observed in the HO group in the PEEK post (8.82 ± 1.11 MPa). Regarding 3D-printed resin post, 30% H_2_O_2_ for 5 min recorded the highest statistically significant bond strength value (13.11 ± 1.61) compared to other groups. Furthermore, there was no significant difference (*p* > 0.05) between C and SB groups. Regarding the milled PEEK post, the 50 μm air abrasion recorded the highest statistically significant (*p* < 0.001) value (23.88 ± 1.66) compared to other groups. Furthermore, there was no significant difference (*p* > 0.05) between C and HO groups.

**Figure 3:**
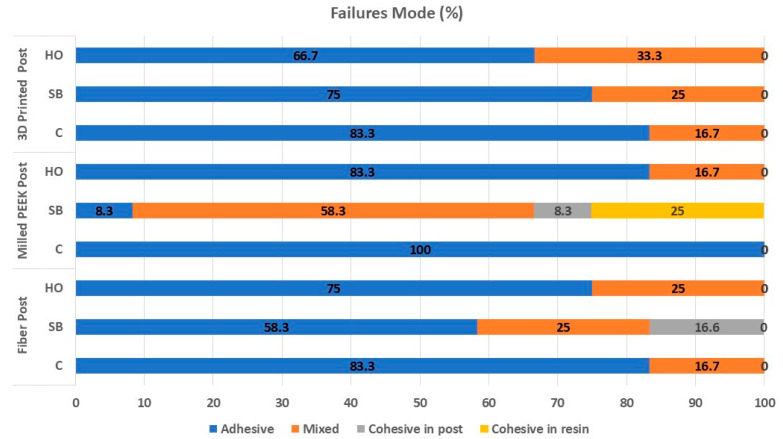
Mode of failures for the different groups.

Mode of failure data are presented in Figure 3. Adhesive failure was the predominant failure type, with an occurrence rate of 70.35%. Mixed failure was noted in 24.07% of the cases, with a significant prevalence in the PEEK within the SB group (58.3%). Cohesive failure was noted in 5.54% of instances, with a significant prevalence in the SB group, occurring at rates of 16.6% in ready-made fiber posts and 33.3% in PEEK posts. With a closer look at the data, in group C, adhesive failure was the predominant type of failure, occurring at a rate of 88.86%, while mixed failure accounted for 11.14%. There were no instances of cohesive failure. In the SB group, adhesive failure was the predominant type of failure, occurring in 49.69% of cases. Conversely, the air particle abrasion in the PK group displayed mixed and cohesive failures as the main types, occurring at rates of 58.3% and 33.3%, respectively. In the HO group, adhesive failure was observed in 75% of cases, while mixed failure occurred in 25%. No instances of cohesive failure were observed.

**Figure 4:**
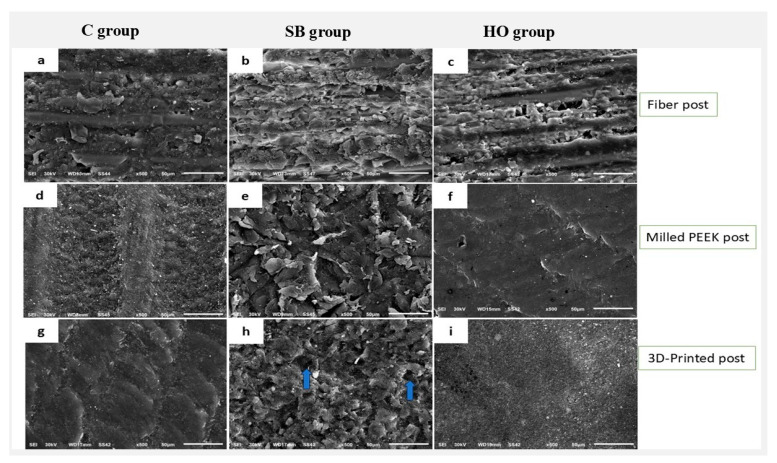
SEM micrographs (500×) (**a**–**c**) glass fiber post; (**d**–**f**) milled PEEK post; (**g**–**i**) 3D-printed resin post. Blue arrow indicates dislodgment of fillers.

The scanning electron microscopy (SEM) examination of the glass fiber post revealed micropores and grooves with superficial glass fibers and resin matrix in group C. Conversely, SEM evaluation of Milled and 3D-printed resin posts revealed polished, smooth, and uniform surfaces (Figure 4a,d,g). The surface morphology of the posts was modified by treatment with air particle abrasions, resulting in all post surfaces exhibiting rough textures with elevations and depressions, which may enhance micromechanical retention in comparison to the untreated post surfaces (Figure 4b,e). Nonetheless, the 3D-printed posts exhibited certain dislodgments of fillers from the surfaces of the resin matrix (blue arrows) (Figure 4h). In the context of hydrogen peroxide surface treatment, RX and CT posts exhibited rough surfaces that offer potential for micromechanical retention when compared to C groups (Figure 4c,i). However, for the PK post, the surface did not alter significantly compared to the control group, as shown in (Figure 4d,f).

## 4. Discussion

One of the most important considerations when using endodontic dental posts is to consider the longevity of a composite resin core restoration depends on establishing an effective bond between the core material and the post material, allowing the interface to effectively transfer stresses during functional loading [6,16,24]. The clinical background of this study was to determine the optimal surface treatment protocol that improves the bonding effectiveness of each post fabrication method, thereby improving adhesion between the post and core material. This study was designed to evaluate and compare the adhesion of a 3D-printed and milled resin post to composite resin core build-up material following different surface treatments. The null hypotheses were rejected due to statistically significant differences observed in surface treatments and post types based on manufacturing methods.

In this study, push-out bond strength was utilized for adhesion testing. It offers a more precise evaluation in comparison to the shear test, since the fracture takes place parallel to the bonding interface instead of transversely, thereby effectively simulating clinical conditions. Additionally, a single bonded post/composite can provide several specimens [16,25,26]. Airborne particle abrasion is a common mechanical surface treatment process. The technique comprises applying alumina particles onto the material surface to enhance surface roughness and increase surface area, therefore enhancing mechanical retention [16,27,28]. The size of particle size is a key variable influencing surface roughness and, hence, adhesion. Particle size commonly ranges from 30 to 110 µm. A 50 µm aluminous particle was utilized in this study since it provide more controlled roughness and provide better results. However, 110 µm has been reported to induce over-abrasion or damage [15,16]. Hydrogen peroxide is widely used in dentistry, particularly for teeth bleaching, and is simple and safe to use. 30% H_2_O_2_ etching is a type of chemical surface treatment that has been reported to provide easy, effective and clinically feasible method for the enhancement of bond strength between fiber posts and core build-up [29].

Single Bond Universal was utilized due to its reflection of the current clinical practices, characterized by its simplicity, versatility, and broad applicability. The adhesive was utilized without a dual-cure activator based on 3M’s guidelines, which state that the adhesive can be used without an activator if it is adequately light-cured [30]. In our study, the adhesive was light-cured prior to the application of dual-cured core material, and care was taken to ensure direct light access [30,31,32]. This could minimize the potential for unreacted acidic monomers to remain at the interface and inhibit polymerization. Furthermore, after the core build-up, specimens were immersed in standardized conditions (37 °C and 100% humidity) for 24 h before bond strength testing allowed sufficient time for the chemical curing of the core material to proceed, thus mitigating any initial polymerization delay caused by acidic pH.

In the current investigation, air particle abrasion significantly improved the push-out bond strength in the RX and PK groups. This could be attributed to the fact that the surface roughness increases, which promotes micromechanical interlocking with the core build-up material. Our findings are consistent with previous research that has demonstrated that the bond strength of glass fiber posts [1,4] and PEEK posts [5,6,16] is substantially enhanced by air particle abrasion in comparison to untreated surfaces. Conversely, the bond strength of the 3D-printed resin post was not significantly improved by air particle abrasion. A previous study reported that air particle abrasion, while roughening the surface of 3D-printed resin polymer, also caused filler exposure. This highlights the potential for air particle abrasion to damage the resin surface and create stress zones [11]. Additionally, our results align with the findings of Lim et al. [31], which indicated that air particle abrasion did not significantly enhance bond strength in the repair of 3D-printed resin using conventional temporary resin. Consequently, caution is recommended when employing air particle abrasion on 3D-printed resins.

It has been reported that using peroxide may affect the adhesive bonding of resin core material due to residual oxygen [8]. This effect was probably not observed due to the lack of residual oxygen in the post structure or maybe due to the limited application time of (5 min). In the current study, 30% H_2_O_2_ for 5 min improved the interfacial bond strength between core materials and both fiber posts (RX group) and 3D-printed resin posts (CT group). This enhancement can be explained by the fact that 30% H_2_O_2_ for 5 min is known to effectively remove the surface layer of the epoxy resin, which increases the surface area and provides additional sites for micromechanical retention [7,8]. This is corroborated by the SEM analysis, which demonstrated the 30% H_2_O_2_’s ability to modify the post surfaces (Figure 4c,i). These findings are in line with Elsaka et al. [32], who reported that the application of 30% H_2_O_2_ for 5 min to the fiber post surfaces enhanced the adhesion to resin cores. On the other hand, 30% H_2_O_2_ for 5 min did not improve the bond strength in the PK group in comparison to the untreated surfaces. This could be attributed to the H_2_O_2_ concentration and/or exposure duration, which was inadequate to enhance the bond strength. Furthermore, PEEK is made of prefabricated CAD/CAM polymers that are industrially polymerized at high pressure and temperature under controlled conditions. As a result, it exhibits significant resistance to surface changes [33,34].

Although the adhesive was adequately light-cured in our study according to manufacturer instructions, the chemical interaction between the acidic monomers in Single Bond Universal and the tertiary amine initiator in the dual-cure core material might interfere with the chemical polymerization at the adhesive–resin interface. Furthermore, this effect combined oxygen inhibition at the adhesive surface may compromise the integrity of the bonding interface, increasing the incidence of adhesive failure.

The clinical relevance of the study was to identify optimal surface treatment protocols that enhance the bonding effectiveness of various post fabrication methods. This will enable manufacturers to supply pre-treated posts, either milled or 3D printed, in pre-sealed sachets, thereby conserving valuable chair time for clinicians. This study has limitations, as it did not consider either the effects of varying pH levels simulating the oral environment or the extended aging periods. Although performing separate printings could reduce the probability of systemic error, any slight changes in environmental conditions may increase variability. Accordingly, utilizing single printing is a limitation in this study, and future investigation should be considered aiming at quantifying batch-to-batch variability in 3D printing for similar applications. The findings of the current study highlight the importance of selecting the proper adhesive system that is chemically compatible with dual-cure material, as well as employing appropriate surface treatments, to ensure the long-term clinical success of adhesive restorations. Improper protocols can affect bonding effectiveness and increase the risk of adhesive failure and debonding over time. Moreover, further in vivo studies are required to emphasize whether the improved performance of the treated posts is consistent with the in vitro findings. The future direction of spectroscopic analysis, including FTIR, should focus on clarifying the polymerization behavior at the adhesive-core interface. Extrapolating laboratory bond strength findings to clinical applications should be done with caution.

## 5. Conclusions

This study aimed to identify the most effective surface treatment protocols that improve bonding efficacy for various post-fabrication methods. Within the limitations of this study, we can draw the following conclusions:

- Air particle abrasion significantly improved the push-out bond strength of milled PEEK posts, while it did not have a similar effect on the 3D-printed resin posts.
- Application of 30% H_2_O_2_ for 5 min to 3D-printed resin post enhanced the adhesion to core build-up material, while it did not have a similar effect on milled PEEK post.
- The manufacturing method of posts, the surface treatments utilized, and their interactions affect the bond strength between posts and the composite resin core build-up material.

## Figures and Tables

**Table 1:** Materials used in the study.

Materials	Product (Composition)	Composition/Manufacturer	Lot. No.	Code
Post	Ready-made-fiber post (Rely X) (Size #3)	- Glass fiber-reinforced (composite, methacrylate resin) - 3M ESPE, St. Paul, MN, USA	255531407	RX
	CAD/CAM milled post Bre CAM Bio HPP (PEEK)	- Poly ether ether ketone, 20wt% titanium dioxide ceramic filler and Aluminum oxide sand (50 µm mean particle size) - Bredent GmbH & Co., Senden, Germany.	56654456	PK
	3D-printed resin post Saremco print CROWNTEC	Bisphenol A diglycidyl methacrylate ethoxylated, trimethyl benzoyl diphenyl phosphine oxide - Saremco Dental AG, Rebstein, Switzerland	E394	CT
Core build-up material	Grandio Core DC (dual-cured composite core material)	- Matrix: Bis-GMA, UDMA resins. Filler: silica/Ba-glass ceramics (77%, wt). Amines, benzoyl peroxide, BHT). - VOCO GmbH, Cuxhaven, Germany.	Z01X78	G_DC_

**Table 2:** Two-way ANOVA table for post type, surface treatment, and their interaction according to push-out bond strength (MPa).

Source of Variations	Sum of Squares	df	Mean Squares	F	*p* Value
Post type	195.790	2	97.895	79.968	*p* < 0.001
Surface treatment	1428.725	2	714.362	583.546	*p* < 0.001
Post type × Surface treatment	1301.254	4	325.314	265.741	*p* < 0.001
Total	22,324.641	108	—	—	—

**Table 3:** Push-out bond strength and modes of failure for the different groups.

Post Type	Surface Treatments		
	C	SB	HO
RX	11.07 ± 0.99 ^Ac^	19.55 ± 0.83 ^Ba^	15.06 ± 1.33 ^Ab^
PK	10.52 ± 0.79 ^Ab^	23.88 ± 1.51 ^Aa^	8.82 ± 1.11 ^Bb^
CT	11.27 ± 0.73 ^Ab^	11.79 ± 0.81 ^Cb^	14.09 ± 1.48 ^Aa^

Mean values represented with the same lowercase letters (row) are not significantly different according to Tukey’s test (*p* > 0.05). Mean values represented with the same uppercase letters (column) are not significantly different according to Tukey’s test (*p* > 0.05).

## Data Availability

The original contributions presented in this study are included in the article. Further inquiries can be directed to the corresponding authors.
